# Obesity Is Associated with Oxidative Stress Markers and Antioxidant Enzyme Activity in Mexican Children

**DOI:** 10.3390/antiox13040457

**Published:** 2024-04-12

**Authors:** Ana Isabel Cota-Magaña, Miguel Vazquez-Moreno, Andrés Rocha-Aguado, Selene Ángeles-Mejía, Adán Valladares-Salgado, Margarita Díaz-Flores, Norma Edith López-Díazguerrero, Miguel Cruz

**Affiliations:** 1 Unidad de Investigación Médica en Bioquímica, Hospital de Especialidades, Centro Médico Nacional Siglo XXI del Instituto Mexicano del Seguro Social, Mexico City 06720, Mexico; ana.isacota@gmail.com (A.I.C.-M.); moreno_029@yahoo.com.mx (M.V.-M.);; 2 Programa de Doctorado en Ciencias Biológicas y de la Salud, Universidad Autónoma Metropolitana, Mexico City 04960, Mexico; 3 Laboratorio de Bioenergética y Envejecimiento Celular, Departamento de Ciencias de la Salud, División de Ciencias Biológicas y de la Salud, Universidad Autónoma Metropolitana Unidad Iztapalapa, Mexico City 09340, Mexico; 4 OOAD Ciudad de México Norte, Unidad de Medicina Familiar No. 23, Instituto Mexicano del Seguro Social, Mexico City 07070, Mexico

**Keywords:** carbonylated proteins, malondialdehyde, catalase, superoxide dismutase, glutathione peroxidase, oxidative stress, antioxidant system, obesity, children, Mexico

## Abstract

The relationship between metabolic disorders and oxidative stress is still controversial in the child population. The present cross-sectional study aimed to analyze the associations between obesity, cardiometabolic traits, serum level of carbonylated proteins (CPs), malondialdehyde (MDA), and the enzyme activity of catalase (CAT), superoxide dismutase (SOD), and glutathione peroxidase (GPx) in children from Mexico City (normal weight: 120; obesity: 81). Obesity resulted in being positively associated with CAT (β = 0.05 ± 0.01, *p* = 5.0 × 10^−3^) and GPx (β = 0.13 ± 0.01, *p* = 3.7 × 10^−19^) enzyme activity. A significant interaction between obesity and sex was observed in MDA and SOD enzymatic activity (*P_MDA_* = 0.03; *P*_SOD_ = 0.04). The associations between obesity, MDA level, and SOD enzyme activity were only significant in boys (boys: *P_MDA_* = 3.0 × 10^−3^; *P_SOD_* = 7.0 × 10^−3^; girls: *p* ≥ 0.79). In both children with normal weight and those with obesity, CP levels were positively associated with SOD enzyme activity (*P_Normal-weight_* = 2.2 × 10^−3^; *P_Obesity_ =* 0.03). In conclusion, in Mexican children, obesity is positively associated with CAT and GPx enzyme activity, and its associations with MDA levels and SOD enzyme activity are sex-specific. Therefore, CP level is positively related to SOD enzyme activity independently of body weight.

## 1. Introduction

Obesity is an important public health problem worldwide because it is considered the main risk factor for insulin resistance, type 2 diabetes, dyslipidemia, and cardiovascular diseases [1,2,3]. In Mexico, the National Health and Nutrition Survey 2022 reported combined prevalence of overweight and obesity in children and adolescents of 37.3% and 41.1%, respectively [4]. Regarding sex, in the child population, the prevalence of girls was 35% and that of boys was 39.4% [4]. In adolescents, the prevalence was 41% in females and 41.1% in males [4].

Obesity is a multifactorial disease that results from the interaction of environmental, social, behavioral, and biological risk factors [5]. Concerning biological risk factors, although it is well known that in utero environment, age, and sex are important determinants for obesity [5], the information is limited and controversial regarding oxidative stress in the child population [6].

Oxidative stress occurs when the oxidative component is favored in an oxidant and antioxidant system [6,7]. Reactive oxygen species, the primary oxidant component in the human body, are produced in the normal metabolic process. However, in excessive adiposity, it has been documented that reactive oxygen species levels increase and exceed the capacity of the antioxidant system [7,8], where the antioxidant enzymes superoxide dismutase (SOD), glutathione peroxidase (GPx), and catalase (CAT) play an important role [9]. Preclinical, clinical, and epidemiological studies have reported the association between metabolic diseases and oxidative stress through an accumulation of products of lipid and protein oxidation and reduced activity of the antioxidant systems [6,10].

In the adult population, protein carbonylation has been reported to be directly correlated with adiposity [11]. In children, serum levels of hydroperoxide (as a marker of systemic oxidative stress), malondialdehyde (MDA, a marker of lipid oxidation), and total oxidant status are higher in obesity than in normal weight [12,13,14,15,16]. These markers were also observed to be positively correlated with body mass index (BMI) [13,14] and cardiometabolic risk factors, such as very low-density lipoprotein, low-density lipoprotein cholesterol (LDL-C), and triglycerides (TG) [15,16].

The levels of the markers of the antioxidant system are not as clear as those of oxidative stress markers. In the child population, while the total antioxidant capacity and the antioxidant enzyme activity of CAT and GPx have been reported to be significantly reduced in children with overweight and obesity in comparison with normal-weight children [13,17,18], the enzymatic activity of SOD has been observed elevated [19,20,21] or without difference [22].

Although there are reports of association trends between oxidative stress markers and metabolic disorders, the status of the antioxidant system remains a topic of ongoing debate, particularly concerning children. Consequently, our objective was to analyze the associations of obesity and cardiometabolic traits with oxidative stress markers and antioxidant enzyme activity in Mexican children.

## 2. Materials and Methods

### 2.1. Study Sample

A total of 201 unrelated Mexican children (120 with normal weight and 81 with obesity) between the ages of 6 and 12 were included in this cross-sectional study. Our study sample corresponds to a case-control design of childhood obesity determined by the Centers for Disease Control (CDC) criteria. The children were recruited in 2017 at the social security centers of the Instituto Mexicano del Seguro Social (IMSS) in Mexico City. The research was approved by the ethics committee of the IMSS (CONBIOETICA-09-CEI-009-20160601; approval number: R-2016-785-097) and was conducted in compliance with the Declaration of Helsinki. Before enrollment in the study, a child’s assent was obtained, and parents or legal guardians approved the participation of children by signing a written informed consent form. Children with acute infection or chronic disease or participating in a weight reduction program were excluded from the study.

### 2.2. Anthropometric Measurements

Body weight and height were measured using a digital scale (Seca, Hamburg, Germany) and a portable stadiometer (Seca 225, Hamburg, Germany), respectively. Body mass index (BMI) was obtained by dividing weight (kg) by the height squared (m)^2^. According to the CDC, BMI were converted to age- and gender-adjusted standard deviation scores (BMI-SDS) using the guidelines from the Centers for Disease Control (CDC) [23]. Additionally, using the CDC 2000 reference, age- and gender-specific BMI percentiles were calculated to classify children with normal weight (BMI between the 5th to 85th percentile) and obesity (BMI at or above the 95th percentile) [24]. Using a mercurial sphygmomanometer (ALPK2, Tokyo, Japan), systolic and diastolic blood pressure (SBP and DBP) were measured for each participant. In a sitting position, measurements were made three times on the right arm at 5 min intervals. The mean of the three readings was considered to carry out all the analyses.

### 2.3. Blood Sample and Biochemical Measurements

Blood samples were collected following a fast for at least 8 h. An ILab 350 Clinical Chemistry System (Instrumentation Laboratory IL, Barcelona, Spain) was used to measure serum levels of fasting plasma glucose (FPG), total cholesterol (TC), high-density lipoprotein cholesterol (HDL-C), LDL-C, and TG. Fasting plasma insulin (FPI) was measured by chemiluminescence (IMMULITE, Siemens, Deerfield, IL, USA), and the homeostatic model assessment of insulin resistance (HOMA-IR) was calculated using the equation by Matthews et al. [25].

### 2.4. Measurement of Oxidative Stress Markers

#### 2.4.1. Malondialdehyde (MDA)

The MDA level was measured as a marker of lipoperoxidation with the thiobarbituric acid reactive substance (TBARS) assay in serum samples. The TBARS assay is based on the reaction between thiobarbituric acid and the product of lipoperoxidation malondialdehyde. We mixed 200 µL of serum, 200 µL of phosphoric acid 0.2 M, 25 µL thiobarbituric acid, and 200 µL deionized water then incubated for 60 min at 90 °C. After this time, 400 µL of butanol was added to the mix and centrifugated at 10,000 rpm for 10 min at 4 °C. The supernatant was separated and measured at 535 nm in a Multiskan FC 357 microplate reader (Thermo Fisher Scientific, Waltham, MA, USA). MDA standard was prepared with 1,1,3,3-tetramethoxypropane and expressed as nM [26]. TBARS concentration was calculated as follows:

$$nmol\;TBARS=\left(Abs\;mean-blank\right)(0.4166)$$

#### 2.4.2. Protein Carbonylation

The carbonyl residues of the oxidized proteins were measured in erythrocyte lysate. The method is based on the reaction of the carbonyl residues with 10 mM 2,4 dinitrophenylhydrazine (DNPH). We added 20 µL of sample and 20 μL of DNPH to a microplate well and incubated for 10 min at 37 °C; after that, we added 10 µL of NaOH 0.6M and incubated again for 10 min at 37 °C. Both times, we maintained constant agitation and samples were covered in light. Once the incubated period was finished, we measured in a Multiskan FC 357 (Thermo Fisher Scientific, Waltham, MA, USA) microplate reader at 340 nm. The results were expressed as μmol of carbonylated proteins/mg of total protein (μmolCPs/mg protein) [27]. Levels of carbonylated proteins were calculated as follows:

$$nmol\;ox=\frac{abs\;mean\times 46.1}{\left[Protein\right]}$$

### 2.5. Measurement of Antioxidant Enzyme Activity

#### 2.5.1. Determination of Total Protein Content

Before antioxidant enzyme activity was measured, the determination of total protein content was performed using the Bradford method. We added 200 µL of Bradford solution (BIORAD) and 800 µL deionized water and 1 µL of serum or erythrocyte lysate and analyzed by spectrophotometry at 595 nm in a Multiskan FC 357 (Thermo Fisher Scientific, Waltham, MA, USA).

#### 2.5.2. Superoxide Dismutase-Specific (SOD) Enzyme Activity

The enzyme activity of SOD was measured in erythrocyte lysate by spectrophotometry assay using the xanthine/xanthine oxidase reaction with nitro blue tetrazolium (NBT) as a detector and a source of superoxide anion and its reaction with NBT. Once NBT is reduced by superoxide anion, it produces formazan dye, which can be measured by spectrophotometry. In the presence of SOD, this reduction reaction cannot be performed due to the dismutation of superoxide anion. For the reaction, we added 145 µL of working solution (50 mM sodium carbonate buffer solution, 0.1 mM xanthine, 0.025 mM NBT, 0.1 mM EDTA), 2.5 µL of sample or PBS as blank (50 mM phosphate buffer, pH 7.5; 1 mM EDTA), and 2.5 µL of xanthine oxidase (2M 0.1 U/mL de ammonium sulfate solution). The reaction was performed in a 96-well plate and read at 540 nm on Multiskan FC 357 (Thermo Fisher Scientific, Waltham, MA, USA). The specific activity was expressed in units per milligram of protein (U/mg protein) [28]. Specific activity was calculated using the formulas listed below:

$$\frac{USOD}{mL}=\frac{\%inhibition}{\left(50\%\right)(mL\;of\;sample)}$$

% of inhibition=∆A560 blank−∆A560 sample

$$∆A560\;sample=\frac{∆Abs\;}{∆t}$$

$$\frac{USOD}{mgprot}=\frac{USOD/ml}{mg\;of\;protein}$$

#### 2.5.3. Catalase-Specific (CAT) Enzyme Activity

CAT enzyme activity was evaluated by measuring the reduction in H_2_O_2_ concentration at 240 nm spectrophotometrically. In brief, a work solution was made with 0.1 M PBS pH 7 and 20 mM H_2_O_2_ solution. A total of 1.5 mL of H_2_O_2_ working solution and 3 µL of sample were added to a quartz cell. It was measured on the Lambda 25 spectrophotometer (Perkin-Elmer Inc., Waltham, MA, USA) at 240 nm every 10 s for 3 min, and the results were expressed in U/mg of protein [28]. CAT-specific activity was calculated as follows:

$$\frac{UCAT}{mL}=\left(\frac{Abs\;initial-Abs\;final}{0.0394}\right)1000$$

#### 2.5.4. Glutathione Peroxidase-Specific (GPx) Enzyme Activity

According to Vazquez-Medina et al. (2006), we analyzed GPx enzyme activity by measuring the continuous reduction in NADPH concentration using H_2_O_2_ as a substrate. In a quartz cell, we mixed PBS (500 mM), EDTA (50 mM), sodium azide (20 mM), glutathione reductase (15 U/mL), NADPH (1.5 mM), reduced glutathione (250 mM), sample, and H_2_O_2_ (10 mM). The absorbance was followed at 340 nm, and the change in absorbance per minute (ΔA340) was calculated. Two blanks, one without H_2_O_2_ and another without sample, were processed simultaneously. The reaction was measured in a Lamba 25 spectrophotometer (Perkin-Elmer, Inc., Waltham, MA, USA). Enzyme activity was expressed in UGPx/mL. One unit of GPx activity is defined as the amount of enzyme that oxidizes 1 µmol of NADPH per minute [28]. The calculation of the specific activity was carried out as follows:

$$\frac{UGPX}{mL}=400*\frac{Blank1-Blank\;2-sample}{6.22}$$

### 2.6. Data Analysis

Differences between children with normal weight and those with obesity for continuous and categorical traits were evaluated using Student’s *t*-test and the Chi-squared test, respectively. The normal distribution of oxidative stress markers and antioxidant enzyme activity was assessed using the Shapiro–Wilk test. A rank-based inverse normal transformation was applied for traits that significantly deviated from normality, and the transformed values were used in the analyses (Appendix A). Linear regression models adjusted for age and sex were used to evaluate the associations of obesity with oxidative stress markers and antioxidant enzyme activity. The interaction effect between sex and obesity on oxidative stress markers and antioxidant enzyme activity was assessed using linear regression models. The associations between cardiometabolic traits, oxidative stress markers, and antioxidant enzyme activity were assessed with Spearman’s correlation analysis, followed by linear regression adjusted for age, sex, and obesity status. Two-sided *p*-values < 0.05 were considered significant in the comparison analysis, and Bonferroni correction for multiple tests was considered in the association analysis [29]. The statistical analyses were conducted using SPSS software (version 22.0, IBM, Armonk, NY, USA).

## 3. Results

### 3.1. General Characteristics of the Study Sample

The general characteristics of the study sample are presented in Table 1. Children with obesity showed higher BMI, WC, SBP, DBP, LDL-C, TG, FPI, and HOMA-IR values than children with normal weight (*p* < 0.05). However, HDL-C levels were lower in the obesity group (*p* = 3.0 × 10^−5^). Sex ratio, age, TC, and FPG did not show significant differences between the groups (*p* > 0.06).

### 3.2. Associations of Obesity with Oxidative Stress Markers and Antioxidant Enzyme Activity

The serum levels of oxidative stress markers and antioxidant enzyme activity in children with normal weight and those with obesity are described in Table 1. While levels of carbonylated proteins, MDA, and SOD enzyme activity did not present significant differences between groups (*p* ≥ 0.09), children with obesity had significantly higher serum enzyme activity of CAT and GPx than children with normal weight (mean differences: Cat = 0.06 mgprot, *p* = 3.0 × 10^−3^; GPx = 0.14 U/mL, *p* = 2.12 × 10^−19^). In the association analysis that was adjusted for age and sex (Table 2), obesity was positively associated with the enzyme activity of CAT (β = 0.05 ± 0.01, *p* = 5.0 × 10^−3^) and GPx (β = 0.13 ± 0.01, *p* = 3.7 × 10^−19^). We did not identify any significant association of obesity with the serum levels of carbonylated proteins, MDA, or SOD enzyme activity (*p* ≥ 0.05, Table 2).

**Figure 1:**
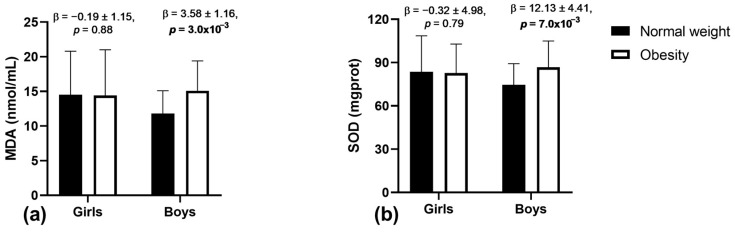
Associations of obesity with (**a**) MDA, and (**b**) SOD enzyme activity in girls and boys separately. Abbreviations: MDA, malondialdehyde; SOD, superoxide dismutase. Data are represented as β ± standard deviation. Sample size: girls: normal weight = 68; obese = 35; boys: normal weight = 52; obese = 46. Analysis by linear regression model adjusted for age. Significant *p*-values (*p* < 0.05) are represented in bold.

We then evaluated if sex modified the association between obesity, oxidative stress markers, and antioxidant enzyme activity (Table 2). We found a significant interaction effect between obesity status and sex on the levels of MDA (β = 0.92 ± 0.43, *p* = 0.03) and SOD enzymatic activity (β = 3.41 ± 1.64, *p* = 0.04). Analyzing girls and boys separately (Figure 1), significant associations of obesity with serum MDA levels (β = 3.58 ± 1.16, *p* = 3.0 × 10^−3^) and SOD enzyme activity (β = 12.13 ± 4.41, *p* = 7.0 × 10^−3^) were only observed for boys. These associations were not significant among girls (MDA: β = −0.19 ± 1.15, *p* = 0.88; SOD: β = −0.32 ± 4.98, *p* = 0.79).

### 3.3. Association between Oxidative Stress Markers and Antioxidant Enzyme Activity

**Figure 2:**
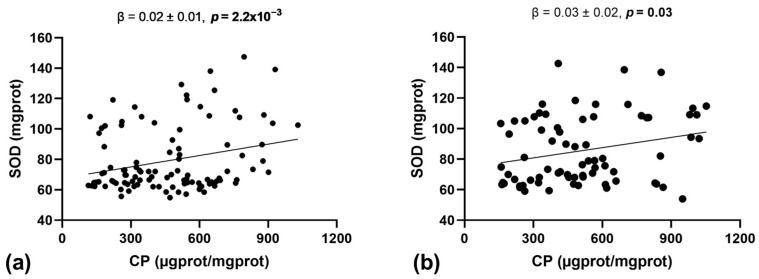
Associations between CPs and SOD in (**a**) children with normal weight (*n* = 120) and (**b**) those with obesity (*n* = 81). Abbreviations: CPs, carbonylated proteins; SOD, superoxide dismutase. Analysis by linear regression model adjusted for age and sex. Significant *p*-values (*p* < 0.05) are represented in bold.

The association analysis between oxidative stress markers and antioxidant enzyme activity is presented in Table 3. A significant correlation was observed between carbonylated protein levels and SOD enzyme activity (Rho = 0.023, *p* = 1.7 × 10^−3^). Additional analysis adjusted for age, sex, and obesity status confirmed this positive association (β = 0.02 ± 0.01, *p* = 1.8 × 10^−4^). The association between carbonylated protein levels and SOD enzyme activity remained significant when it was analyzed separately for children with normal weight and those with obesity (Figure 2; normal weight: β = 0.02 ± 0.01, *p* = 2.2 × 10^−3^; obese: β = 0.03 ± 0.02, *p* = 0.03). We did not identify any other significant association between oxidative stress markers and antioxidant enzyme activity (Table 3; *p* ≥ 0.36).

### 3.4. Associations between Cardiometabolic Traits, Oxidative Stress Markers, and Antioxidant Enzyme Activity

The association analysis between cardiometabolic traits, oxidative stress markers, and antioxidant enzyme activity is shown in Table 4. We found some nominal associations, e.g., diastolic blood pressure with carbonylated proteins (*p* = 0.04), FPG with CAT enzyme activity (*p* = 0.03), FPI with SOD (*p* = 2.0 × 10^−3^) and GPx enzyme activity (*p* = 0.02), and HOMA-IR with SOD (*p* = 3.0 × 10^−3^) and GPx enzyme activity (*p* = 0.03). None of these associations remained significant after Bonferroni correction for multiple tests (Table 4, *p* < 1.1 × 10^−3^).

## 4. Discussion

The present study evaluated the associations of obesity and cardiometabolic traits with oxidative stress markers and antioxidant enzyme activity in a sample of Mexican children. Children with obesity had higher values for SBP, DBP, LDL-C, TG, FPI, and HOMA-IR and lower HDL-C compared to normal-weight children, which could be related to a higher risk of developing metabolic complications and cardiovascular disease. Additionally, our results show (a) a positive association of obesity with the enzyme activity of CAT and GPx, (b) a sex-specific association of obesity with lipoperoxidation and the enzyme activity of SOD, and (c) a positive association between carbonylated protein levels and SOD enzyme activity, independently of obesity status.

The enzymatic activity of SOD, CAT, and GPx plays a crucial role in first-line defense, as an antioxidant, neutralizing free radicals and peroxides generated in lipophilic environments so that they are efficiently eliminated in hydrophilic domains, thus protects cells and tissues from oxidative stress [30]. SOD is mainly active in cell membranes or mitochondria, catalyzing the dismutation of two molecules of superoxide anion (∗O_2_) to hydrogen peroxide (H_2_O_2_) and molecular oxygen (O_2_), making the superoxide anion less harmful [30]. CAT catalyzes the reduction of H_2_O_2_ to water and molecular oxygen that is generated by SOD, mainly in cellular peroxisomes, preventing cellular damage [30]. GPx operates in lipophilic environments and removes H_2_O_2_ and other fat-soluble peroxides, using glutathione as a cofactor to neutralize peroxides and convert them to water [30].

Until now, there has been no consensus regarding the direction (negative/positive) of the associations between childhood obesity and the enzyme activity of SOD, CAT, and GPx. In our study, children with obesity showed significantly higher CAT and GPx enzyme activity than children with normal weight. The positive associations between obesity and the enzyme activity of CAT and GPx that we evidenced in this study are consistent with previous reports. Studies conducted with Asian [31] and European [32] children have observed higher GPx enzyme activity among children with obesity than in those with normal weight. In the case of CAT, a previous study conducted with Asian children reported that the gene expression of CAT, which is mainly related to CAT enzyme activity, was higher among children with obesity than those with normal weight [33]. The gene expression and enzymatic activity of CAT have been reported to be regulated by oxidative stress. Animal models have reported overexpression and high enzymatic activity in response to a high-fat diet, which results in the prevention of oxidative damage [34]. Due to both enzymes being involved in cellular defense against oxidative damage, one possible explanation for these associations that has been suggested is an adaptative process to respond to the progression of oxidative stress in obesity [32,35]. The literature has described that age, the degree of adiposity, and metabolic affectation may contribute to variations in the antioxidant status [33]. In this regard, a previous study reported that children with obesity without insulin resistance increased in total erythrocyte glutathione-equivalent enzyme activity during a glucose tolerance test, a response similar to normal-weight children [15]. In this experiment, the total erythrocyte glutathione equivalent enzyme activity did not change among children with obesity and insulin resistance. In line with this hypothesis, Mohseni et al. (2018) reported that higher CAT gene expression could reduce the metabolic complications related to obesity due to its positive correlations with BMI, LDL-C, HOMA-IR, and SBP among Iranian children [33].

Concerning the sex-specific associations of obesity with MDA and the enzyme activity of SOD that we found, previous studies on children have evidenced that girls could be more resistant to developing metabolic complications related to obesity than boys [36]. Furthermore, in animal models, it has been observed that female rats fed with a high-fat diet show less insulin resistance than males [37]. It has also been reported that the responses of oxidative stress markers and antioxidant enzyme activity to chronic administration of a high-fat diet are more sensitive in male rats than in females [38,39]. This evidence suggests that the oxidative stress markers and antioxidant enzyme responses may be related to sexual hormones [31]. The observed differences in associations between boys and girls underscore the importance of considering gender when researching and managing obesity and its metabolic implications. They might suggest differentiated strategies in metabolic health intervention between boys and girls.

Carbonylated proteins are often considered an important marker of oxidative stress because they are an irreversible modification that can affect protein function and are related to various pathological states. On the other hand, SOD is a crucial antioxidant enzyme, which is fundamental in detoxifying reactive oxygen species, thus protecting against oxidative stress. In this way, the positive association between carbonylated protein levels and SOD enzyme activity, independently of obesity status, could support the hypothesis regarding the adaptation process to respond to the progression of oxidative stress [32,35] that we cited in previous lines. Even the age of the sample in our study further supports the idea that the degree of adiposity and metabolic impairment can contribute to variations in the oxidative stress response process [40]. In our results, neither carbonylated protein levels nor SOD enzyme activity presented a direct association with obesity or any cardiometabolic trait. In contrast, a recent study on Mexican adults reported that SOD enzymatic activity was significantly decreased in overweight and hypertensive subjects. At the same time, carbonyl groups in proteins were significantly increased in hypertensive subjects [41]. Understanding the biological implications of this relationship and its impact on oxidative stress and metabolic health could provide insights into potential therapeutic targets or strategies to manage or prevent associated health problems. For this reason, analyzing the causality and directionality of this relationship will be crucial to establishing whether modulating SOD activity (or reducing carbonylated protein levels) could be beneficial in clinical or therapeutic settings.

The association between insulin resistance markers and the activity of antioxidant enzymes has been little described, and the few studies that exist report contradictory results. In the case of the positive associations of fasting plasma insulin and HOMA-IR with SOD enzyme activity observed in this study, the results are in line with the previous report on a cohort of children from Iran with normal weight or obesity, where the gene expression of Mn-SOD was founded in a positive correlation with serum insulin levels and HOMA-IR [42]. However, in normal-weight Korean children, HOMA-IR was observed in a negative association with SOD enzyme activity. In this regard, the molecular mechanisms to explain this association are not very clear. Nevertheless, previous studies have proposed that the metabolic complications of obesity, such as insulin resistance, may be considered as an independent risk factor of ROS production, which turns out to be a particular stimulant of the antioxidant response [19].

The nominal associations between cardiometabolic traits, oxidative stress markers, and antioxidant enzyme activity observed in this study may point out a possible connection between oxidative stress, antioxidant defenses, and cardiometabolic health, which could be a reason to develop further investigation. In this way, these associations could be driven by shared biological pathways or mechanisms. For example, oxidative stress is known to be involved in the pathogenesis of insulin resistance [43], and antioxidants are crucial in mitigating the effects of oxidative stress and potentially protecting against associated metabolic dysregulations. While the present data do not show robust statistical associations, exploring these pathways and mechanisms in further studies could provide valuable insights.

Our observational results could support the proposal that the increase in the antioxidant activity of CAT, GPx, and SOD enzymes may be due to an adaptive response to oxidative stress, which could also be influenced by mechanisms related to sex hormones. However, it is essential to continue doing cross-sectional studies in different populations, such as children, young adults, and older adults, as well as follow-up studies to generate evidence that supports or refutes this proposal. Exploring these relationships and associations in other populations or age groups could also be beneficial to determine if they are consistent across different demographics or developmental stages.

Although our study is one of the first pieces of evidence of associations between childhood obesity, oxidative stress markers, and antioxidant enzyme activity in the Mexican population, we recognize that it presents some limitations. We are aware that the lack of consistency in the biological samples for the measurement of antioxidant enzymes, oxidative stress markers, and cardiometabolic traits can be disconcerting. However, in the literature, it has been described that the erythrocyte is a good biological sample to measure the antioxidant activity of the enzyme system because it exhibits a self-sustained activity of antioxidant defense enzymes, preventing oxidative cellular damage [44]. Regarding the measurements of MDA, it is considered the most commonly used biomarker that reflects lipid peroxidation levels in biological fluids. In the present work, we measured it in serum samples because in obesity, there is greater production of triglycerides and a decrease in their clearance in serum [45]. In this way, the literature describes that fatty acids associated with triglycerides are more prone to oxidation [46]. Finally, metabolic markers related to obesity were measured in serum because it is where they have been widely studied and related to the different molecular processes involved in the pathophysiology of obesity. Another point to improve in this work is the measurement of MDA. Although direct measurement of MDA has been described to be more reliable with sophisticated methods such as the liquid chromatography–mass spectrometry method [47], in the present study, we measured TBARS to evaluate lipid peroxidation because it is a technique widely used in the scientific literature. Compared to chromatography, the TBARS method is relatively simple, making it easy to implement in clinical and research laboratories without the need for specialized equipment [42]. Another limitation of our study is the absence of data regarding the potential influence of puberty status, a factor that is recommended to be investigated in subsequent studies. Moreover, we focused solely on children, meaning we must determine if the results also apply to adults. Additionally, our study was performed with a small sample size, which encourages the development of studies with larger sample sizes in order to replicate the associations described in this work. Due to our small sample size, we could not explore the associations among girls and boys with and without obesity separately, which could have provided a clearer understanding of the influence of sexual hormones on these associations. Another important limitation of our study is the lack of data related to food intake. It is well known that obesity is mainly related to environmental risk factors, such as a diet characterized by high quantities of refined sugars and saturated fats [48]. In this way, it is of interest to emphasize that, although dietary intake data were not analyzed in this study, the latest National Health and Nutrition Survey in Mexico (2022) reported that 92.9% of schoolchildren in Mexico consume sweetened beverages, which are highly related with childhood obesity [49].

## 5. Conclusions

Our results for Mexican children evidence that obesity is associated with an increase in CAT and GPx enzyme activity and that its associations with lipid peroxidation and SOD enzyme activity are sex-specific. Independently of obesity status, our data also showed a positive association between carbonylated protein levels and SOD enzyme activity, which could be related to an adaptation process to respond to the progression of oxidative stress. While our study offers an initial look and introduces new perspectives regarding obesity and oxidative stress in Mexican children, it also paves the way for future research. Subsequent studies will need to delve deeper and explore the unanswered questions and limitations presented here. Doing so will not only broaden our academic and clinical knowledge, but it might also improve preventive and therapeutic strategies to address the complications associated with childhood obesity and its metabolic consequences. Thus, while providing new insights, our research also serves as a driver for more in-depth and extensive research in the future.

## Figures and Tables

**Table 1:** General characteristics of the study sample.

Traits	Normal Weight *n* = 120	Obese *n* = 81	*p*-Value
Girls/Boys, *n* (%)	68 (56.7)/52 (43.3)	35 (43.2)/46 (56.8)	0.06
Age, years	8.63 ± 1.58	8.77 ± 1.54	0.52
Body mass index, kg/m^2^	16.86 ± 1.75	24.15 ± 2.49	**1.48 × 10^−47^**
Systolic blood pressure, mmHg	100.14 ± 9.01	109.53 ± 10.34	**1.08 × 10^−10^**
Diastolic blood pressure, mmHg	62.32 ± 7.37	69.19 ± 8.83	**1.04 × 10^−8^**
Total cholesterol, mg/dL	157.53 ± 28.58	164.25 ± 28.35	0.10
High-density lipoprotein cholesterol, mg/dL	52.54 ± 11.63	45.70 ± 10.33	**3.0 × 10^−5^**
Low-density lipoprotein cholesterol, mg/dL	89.95 ± 20.93	99.32 ± 19.71	**0.02**
Triglycerides, mg/dL	86.95 ± 41.92	130.72 ± 62.87	**2.03 × 10^−7^**
Fasting plasma glucose, mg/dL	77.67 ± 6.71	76.93 ± 6.43	0.444
Fasting plasma insulin, μU/mL	5.27 ± 3.32	10.27 ± 7.01	**3.71 × 10^−8^**
HOMA-IR	1.03 ± 0.69	1.95 ± 1.44	**6.86 × 10^−7^**
Carbonylated proteins, µgprot/mgprot	484.22 ± 246.05	514.69 ± 253.24	0.39
malondialdehyde, nmol/L	13.33 ± 5.36	14.83 ± 6.97	0.09
Superoxide dismutase, mgprot	79.79 ± 21.94	85.05 ± 22.08	0.11
Catalase, mgprot	0.12 ± 0.10	0.18 ± 0.11	**3.0 × 10^−3^**
Glutathione peroxidase, U/mL	0.05 ± 0.06	0.19 ± 0.11	**2.12 × 10^−19^**

The difference in sex ratios was analyzed using the Chi-squared test. Differences in means were analyzed using Student’s *t*-tests. Abbreviations: HOMA-IR, homeostatic model assessment of insulin resistance. Significant *p*-values (*p* < 0.05) are represented in bold.

**Table 2:** Associations of obesity with oxidative stress markers and antioxidant enzyme activity.

Trait/Sample	Main Effect (a)		Interaction (b)	
	Obesity	*p*-Value	Obesity × Sex	*p*-Value
CPs (µgprot/mgprot)	33.77 ± 36.47	0.35	6.26 ± 18.13	0.73
MDA (nmol/L)	1.71 ± 0.89	0.05	0.92 ± 0.43	**0.03**
SOD (mgprot)	5.79 ± 3.34	0.08	3.41 ± 1.64	**0.04**
CAT (mgprot)	0.05 ± 0.01	**5.0 × 10^−3^**	0.01 ± 0.01	0.95
GPx (U/mL)	0.13 ± 0.01	**3.7 × 10^−19^**	0.01 ± 0.01	0.51

Data are expressed as beta value ± standard error (*p*-value). Abbreviations: CPs, carbonylated proteins; MDA, malondialdehyde; SOD, superoxide dismutase; CAT, catalase; GPx, glutathione peroxidase. Analysis by linear regression model adjusted for (a) age and sex, and (b) age. Significant *p*-values (main effect: *p* < 0.01 after Bonferroni correction [0.05/5] and interaction: *p* < 0.05) are reported in bold.

**Table 3:** Association between oxidative stress markers and antioxidant enzyme activity.

Trait	SOD (mgprot)	CAT (mgprot)	GPx (U/mL)
CPs (µgprot/mgprot)	0.23 **(1.7 × 10^−3^)**	0.07 (0.35)	0.04 (0.58)
MDA (nmol/L)	0.07 (0.36)	0.04 (0.56)	−0.02 (0.78)

Data are represented as Rho (*p*-value). Abbreviations: CPs, carbonylated proteins; MDA, malondialdehyde; SOD, superoxide dismutase; CAT, catalase; GPx, glutathione peroxidase. Analysis by Spearman correlation. Significant *p*-values (*p* < 8.3 × 10^−3^ after Bonferroni correction [0.05/6]) are reported in bold.

**Table 4:** Associations between cardiometabolic traits, oxidative stress markers, and antioxidant enzyme activity.

Trait	CPs	Lip	SOD	CAT	GPx
Systolic blood pressure, mmHg	0.06 (0.38)	0.08 (0.32)	0.09 (0.32)	−0.01 (0.81)	−0.07 (0.33)
Diastolic blood pressure, mmHg	0.16 (0.04)	0.07 (0.31)	0.08 (0.31)	0.05 (0.50)	−0.09 (0.25)
Total cholesterol, mg/dL	0.12 (0.13)	−0.03 (0.67)	0.11 (0.16)	0.04 (0.55)	0.01 (0.95)
High-density lipoprotein cholesterol, mg/dL	0.10 (0.18)	−0.03 (0.64)	0.14 (0.07)	0.02 (0.77)	0.12 (0.11)
Low-density lipoprotein cholesterol, mg/dL	0.09 (0.25)	−0.01 (0.82)	0.05 (0.46)	0.04 (0.59)	−0.04 (0.62)
Triglycerides, mg/dL	0.04 (0.57)	−0.06 (0.44)	0.03 (0.66)	0.01 (0.99)	−0.02 (0.76)
Fasting plasma glucose, mg/dL	0.03 (0.65)	0.01 (0.82)	0.02 (0.76)	−0.17 (0.03)	−0.05 (0.51)
Fasting plasma insulin, μU/mL	0.09 (0.24)	−0.10 (0.17)	0.24 **(2.0 × 10^−3^)**	−0.12 (0.12)	−0.18 (0.02)
HOMA-IR	008 (0.32)	−0.10 (0.18)	0.23 **(3.0 × 10^−3^)**	−0.13 (0.08)	−0.17 (0.03)

Data are represented as Rho (*p*-value). Abbreviations: CPs, carbonylated proteins; LIP, lipoperoxidation; SOD, superoxide dismutase; CAT, catalase; GPx, glutathione peroxidase. Analysis by Spearman rank correlation adjusted for age, sex, and obesity status. Significant *p*-values (*p* < 1.1 × 10^−3^ after Bonferroni correction [0.05/40]) are reported in bold.

## Data Availability

The data set generated and analyzed in the current study is available from the corresponding author upon reasonable request.

## Appendix A

Normality distribution evaluation.

**Table:**

Trait	Shapiro–Wilk Test (*p*-Value)	
	Untransformed	Transformed
Carbonylated proteins (µgprot/mgprot)	7.4 × 10^−6^	0.550
Lipoperoxidation (nmol/L)	2.3 × 10^−15^	0.991
Superoxide dismutase (mgprot)	3.2 × 10^−10^	0.583
Catalase (mgprot)	7.7 × 10^−12^	0.813
Glutathione peroxidase (U/mL)	7.3 × 10^−14^	0.276

Shapiro–Wilk tests were performed on metabolic outcomes before and after rank transformation to determine whether they were normally distributed.
